# Increasing Incidence of Nontuberculous Mycobacteria, Taiwan, 2000–2008

**DOI:** 10.3201/eid1602.090675

**Published:** 2010-02

**Authors:** Chih-Cheng Lai, Che-Kim Tan, Chien-Hong Chou, Hsiao-Leng Hsu, Chun-Hsing Liao, Yu-Tsung Huang, Pan-Chyr Yang, Kwen-Tay Luh, Po-Ren Hsueh

**Affiliations:** Cardinal-Tien Hospital, Taipei, Taiwan (C.-C. Lai); Chi-Mei Medical Center, Tainan, Taiwan (C.-K. Tan); National Taiwan University College of Medicine, Taipei (C.-H. Chou, H.-L. Hsu, Y.-T. Huang, P.-C. Yang, K.-T. Luh, P.-R. Hsueh); and Far Eastern Memorial Hospital, Taipei (C.-H. Liao)

**Keywords:** Nontuberculous mycobacteria, tuberculosis and other mycobacteria, incidence, Taiwan, dispatch

## Abstract

To assess the species distribution and epidemiologic trends of nontuberculous mycobacteria, we examined isolates from patients in Taiwan. During 2000–2008, the proportion increased significantly from 32.3% to 49.8%. Associated disease incidence increased from 2.7 to 10.2 cases per 100,000 patients. *Mycobacterium avium* complex and *M. abscessus* were most frequently isolated.

In Taiwan, the incidence of tuberculosis (TB) remains high despite advances in the antimycobacterial therapy and implementation of well-known TB control measures (*1*). Therefore, patients with acid-fast bacilli (AFB)–positive specimens, especially respiratory samples, are generally presumed to be infected with *Mycobacterium tuberculosis* and are treated with antituberculosis agents and placed in isolation rooms. Increased isolation of nontuberculous mycobacteria (NTM) causing mycobacterial diseases (*2**,**3*) implies that more patients with AFB-positive samples have received inappropriate or unnecessary empirical antituberculous treatment.

To help clinicians determine whether to initiate antituberculous treatment, mycobacterial laboratories should provide percentages of NTM isolates among specimens with positive AFB. However, the number and species of NTM from clinical specimens are increasing in the clinical laboratory, and the distribution of NTM species varies by geographic area (*4*). We thus sought to assess the species distribution of NTM isolates from various clinical specimens and to elucidate the epidemiologic trends of NTM isolates and diseases over a 9-year period in Taiwan.

## The Study

To assess prevalence of NTM isolates, we used the database of the National Taiwan University Hospital mycobacterial laboratory and prepared clinical specimens for mycobacteria cultures according to recommended guidelines (*5*). Before 2001, specimens were spread onto Lowenstein-Jensen slants and Middlebrook 7H11 medium (BBL; Becton, Dickinson and Company Diagnostic Instrument Systems, Sparks, MD, USA); since late 2001, they have been spread onto Lowenstein-Jensen slants and tested by the fluorometric BACTEC system (BACTEC *Mycobacterium* Growth Indicator Tube 960 system; Becton, Dickinson and Company). NTM were identified to the species level by using conventional biochemical methods. Some unidentified NTM species were further confirmed by sequencing of their 16S rRNA gene (1,464 bp) by using 2 primers (8FPL and 1492) as previously described (*6**,**7*)*.* Diseases caused by NTM were defined according to previous description (*4*). Annual incidence and isolation ratio of TB and NTM over time were evaluated by the Cochran-Armitage test for trend. We considered p<0.05 to be significant. To define NTM disease incidence and prevalence, for patients who had isolates over successive years during the study period we included only the year in which they first appeared in the laboratory database.

From January 2000 through December 2008, the laboratory received 283,394 clinical samples for mycobacterial culture. From a total of 23,499 (8.3%) specimens with positive mycobacterial culture results, *M. tuberculosis* were isolated from 14,295 (5.0%) specimens from 3,695 patients and NTM were isolated from 9,204 (3.2%) specimens from 4,786 patients. The trends of decreasing *M. tuberculosis* isolation and increasing NTM isolation were significant (p<0.05 for each) (Figure, panel A).

**Figure:**
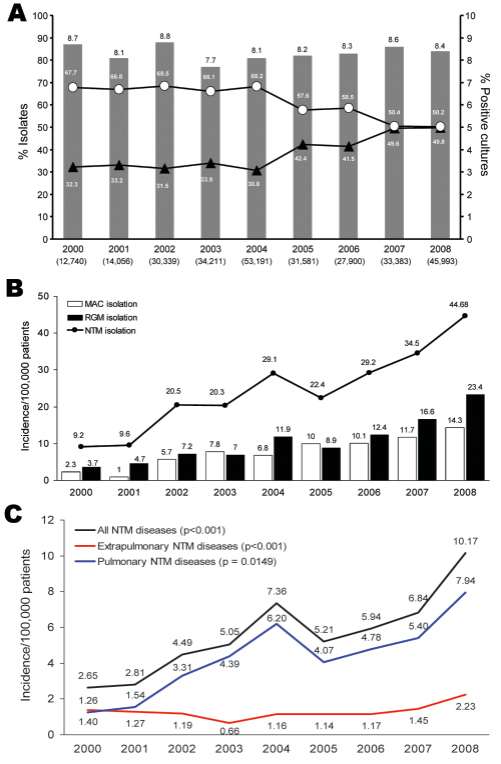
Incidence data for clinical samples submitted for mycobacterial culture, National Taiwan University Hospital, Taipei, Taiwan, January 2000–December 2008. A) Annual number and rate of isolates of nontuberculous mycobacteria (NTM) (triangles) and *Mycobacterium tuberculosis* (circles). B) Annual incidence of isolates of NTM, *M. avium* complex (MAC), and rapidly growing mycobacteria (RGM). C) Annual incidence of NTM disease, pulmonary disease, and extrapulmonary disease.

Among the 9,204 NTM isolates, *M. avium* complex (MAC, 30.0%) were the most frequently isolated organisms, followed by *M. abscessus* (17.5%), *M. fortuitum* complex (13.0%), *M. chelonae* complex (9.6%), *M. kansasii* (5.6%), and *M. gordonae* (5.5%) (Table). Among NTM isolates from the 4,786 patients, prevalent species were MAC (31.7%), *M. fortuitum* complex (18.2%), *M. abscessus* (17.2%), *M. gordonae* (11.6%), *M. chelonae* complex (8.2%), and *M. kansasii* (6.0%) (Table). In addition, some rare isolates such as *M. celatum* (n = 3), *M conceptionene* (n = 3), *M. neoaurum* (n = 2), *M. arupense* (n = 1), *M. mageritense* (n = 1), *M. asiaticum* (n = 1), and *M. immunogenum* (n = 1) were identified.

**Table:** Frequently isolated nontuberculous mycobacteria, Taiwan

Bacterial species	Year, no. isolates/no. patients (% isolates/% patients)								
	2000	2001	2002	2003	2004	2005	2006	2007	2008
*Mycobacterium avium* complex	102/50 (28.5/25.3)	38/23 (10.1/10.1)	244/134 (28.7/27.6)	328/154 (36.7/38.3)	340/158 (25.6/23.2)	449/246 (40.1/44.7)	314/199 (32.7/34.6)	375/242 (26.3/33.8)	571/309 (29.7/32.1)
*Mycobacterium abscessus*	60/36 (16.8/18.2)	94/51 (24.9/23.3)	123/69 (14.6/14.2)	139/63 (15.6/15.6)	163/85 (12.3/12.5)	181/91 (16.5/16.5)	199/98 (20.7/17.0)	250/140 (16.9/19.6)	400/191 (20.8/19.8)
Total	358/198	378/219	841/485	893/402	1,327/679	1,098/550	961/575	1,424/716	1,924/962

Annual incidences are shown in the Figure, panels B and C. During the study period, the proportions of NTM and rapidly growing mycobacteria among all mycobacteria isolated increased significantly from 32.3% to 49.8% (p<0.05) and from 3.7% to 23.4% (p<0.05), respectively. Incidence of diseases caused by NTM also increased (p<0.0001) from 2.7 to 10.2 per 100,000 inpatients and outpatients. The incidences of pulmonary and extrapulmonary NTM infection significantly increased from 1.26 to 7.94 per 100,000 inpatients (p<0.0001) and from 1.4 to 2.23 per 100,000 outpatients (p = 0.0194), and the increase of all NTM diseases was predominately in pulmonary diseases. During the 9 years, 1,105 patients had NTM diseases; most commonly pulmonary disease (n = 894, 76.8%), followed by soft tissue infection (n = 122, 11.4%), disseminated infection (n = 79, 7.1%), and peritonitis (n = 19, 1.7%). MAC was the most common cause of pulmonary infection (n = 342, 40.3%) and disseminated infection (n = 56, 70.1%); *M. abscessus* was the most common cause of skin and soft tissue infection (n = 46, 37.7%).

## Conclusions

During the 9-year period, NTM accounted for 39.2% of positive mycobacterial cultures and increased significantly. In concordance with the increased incidence of NTM isolations, incidence of NTM diseases also increased significantly. The increase of NTM isolations since 2002 might have resulted from use of the BACTEC system. This apparent increase in NTM disease could also be attributed to increasing vigilance and awareness of these bacteria as human pathogens, improved methods of detection, or more immunocompromised hosts (e.g., as a result of tumor necrosis factor inhibitors, human interleukin 1 receptor antagonists, and anti-CD20 antibodies) (*1*,*8*).

Prevalence of mycobacteria species responsible for different diseases varies markedly by geographic region. In the United States and Japan, MAC and *M. kansasii* are the most common species (*4*), whereas in England and Scotland, *M. kansasii* and *M. malmoens*e, respectively, are the most common (*9*). Our study showed that MAC was the most common NTM species in Taiwan, followed by *M. abscessus.* The most common organism in localized pulmonary infection and disseminated infection was MAC, and *M. abscessus* predominated in skin and soft tissue infection and lymphadenitis, consistent with findings of a previous study in Taiwan (*2*)*.* Thus, *M. abscessus* deserves as much attention as MAC, especially for extrapulmonary NTM disease, in Taiwan.

Identification of clinical isolates beyond the genus level is crucial because NTM species differ in the clinical spectrum of the diseases they cause and in their susceptibility to antimicrobial drugs. Previous studies have demonstrated that the rare strains identified in this study are pathogenic and cause human infections, e.g., 1 case of catheter-related bloodstream infection caused by *M. neoaurum*, 1 case of pulmonary infection caused by *M. celatum*, and 2 cases of soft tissue infection caused by *M. conceptionene* and *M. arupense* (*10*–*13*). In addition, in this study *M. mageritense* and *M. immunogenum* were the causative agents for pulmonary infection in an adult and submandibular abscess in a child from Taiwan, respectively.

One study limitation was lack of a quantifiable denominator, which is critical for understanding the epidemiology of an illness. True population-based inferences about NTM epidemiology are usually impossible to conclude from a study of only hospital inpatients and outpatients. Although the hospital has a reference mycobacteriology laboratory and is a major referral center in Taiwan, how the study sample may have affected the results or the approximate size of its catchment of patients remains unknown. During the study period, an increasing percentage of patients (≈20% in 2008) was referred from other hospitals in different parts of Taiwan. However, because this study was conducted in a tertiary-care center in northern Taiwan, these findings might not reflect the overall situation in Taiwan.

As prevalence and incidence of NTM increases, clinicians in Taiwan should consider NTM as a possible cause of TB-like disease. Accurate species identification is imperative before proper treatment can be determined for diseases caused by the diversity of NTM species. Further studies of clinical isolates are also needed to understand the spectrum of disease caused by these rare pathogens.
